# Continuation of emtricitabine/lamivudine within combination antiretroviral therapy following detection of the M184V/I HIV‐1 resistance mutation

**DOI:** 10.1111/hiv.12829

**Published:** 2020-01-11

**Authors:** OT Stirrup, D Asboe, A Pozniak, CA Sabin, R Gilson, NE Mackie, A Tostevin, T Hill, DT Dunn, the UK HIV Drug Resistance Database and the UK Collaborative HIV Cohort, David Chadwick, Duncan Churchill, Duncan Clark, Simon Collins, Valerie Delpech, Samuel Douthwaite, Esther Fearnhill, Kholoud Porter, Christophe Fraser, Anna Maria Geretti, Rory Gunson, Antony Hale, Stéphane Hué, Linda Lazarus, Andrew Leigh‐Brown, Tamyo Mbisa, Chloe Orkin, Eleni Nastouli, Deenan Pillay, Andrew Phillips, Erasmus Smit, Kate Templeton, Peter Tilston, Erik Volz, Ian Williams, Hongyi Zhang, Keith Fairbrother, Justine Dawkins, Siobhan O’Shea, Jane Mullen, Alison Cox, Richard Tandy, Tracy Fawcett, Mark Hopkins, Clare Booth, Ana Garcia‐Diaz, Lynne Renwick, Matthias L. Schmid, Brendan Payne, Jonathan Hubb, Simon Dustan, Stuart Kirk, Amanda Bradley‐Stewart, Jonathan Ainsworth, Sris Allan, Jane Anderson, Ade Apoola, Ian Fairley, Ashini Fox, Mark Gompels, Phillip Hay, Rajesh Hembrom, Margaret Johnson, Sophie Jose, Stephen Kegg, Clifford Leen, Dushyant Mital, Mark Nelson, Hajra Okhai, Adrian Palfreeman, Ashley Price, Frank Post, Jillian Pritchard, Achim Schwenk, Anjum Tariq, Roy Trevelion, Andy Ustianowski, John Walsh, Nadine van Looy, Janet Lynch, James Hand, Stuart Tilbury, Elaney Youssef, Richard Daly, Sundhiya Mandalia, Sajid Munshi, Ade Adefisan, Chris Taylor, Zachary Gleisner, Fowzia Ibrahim, Lucy Campbell, Kirsty Baillie, Sheila Miller, Chris Wood, Mike Youle, Nicky Mackie, Alan Winston, Jonathan Weber, Farhan Ramzan, Mark Carder, Andrew Kerr, David Wilks, Sheila Morris, Sue Allan, Adam Lewszuk, Victoria Ogunbiyi, Sue Mitchell, Christopher Hunt, Olanike Okolo, Benjamin Watts, Sarah Russell‐Sharpe, Olatunde Fagbayimu, Debra Brain, Liz Radford, Sarah Milgate, Shirley Cumming, Claire Atkinson, Annie Rose, Jeanette Smith, Cynthia Murphy, Ilise Gunder, Howard Gees, Gemma Squires, Laura Anderson, Serena Mansfield, Lee Tomlinson, Christine LeHegerat, Roberta Box, Tom Hatton, Doreen Herbert, Ian McVittie, Victoria Murtha, Laura Shewan, Zak Connan, Luke Gregory, Kathleen Holding, Victoria Chester, Trusha Mistry, Catherine Gatford

**Affiliations:** ^1^ Institute for Global Health University College London London UK; ^2^ Chelsea and Westminster Hospital London UK; ^3^ London School of Hygiene & Tropical Medicine London UK; ^4^ CNWL Mortimer Market Centre London UK; ^5^ Imperial College Healthcare NHS Trust London UK

**Keywords:** emtricitabine, HIV, lamivudine, M184I, M184V

## Abstract

**Objectives:**

The aim of the study was to investigate whether lamivudine (3TC) or emtricitabine (FTC) use following detection of M184V/I is associated with better virological outcomes.

**Methods:**

We identified people with viruses harbouring the M184V/I mutation in UK multicentre data sets who had treatment change/initiation within 1 year. We analysed outcomes of viral suppression (< 200 HIV‐1 RNA copies/mL) and appearance of new major drug resistance mutations (DRMs) using Cox and Poisson models, with stratification by new drug regimen (excluding 3TC/FTC) and Bayesian implementation, and estimated the effect of 3TC/FTC adjusted for individual and viral characteristics.

**Results:**

We included 2597 people with the M184V/I resistance mutation, of whom 665 (25.6%) were on 3TC and 458 (17.6%) on FTC. We found a negative adjusted association between 3TC/FTC use and viral suppression [hazard ratio (HR) 0.84; 95% credibility interval (CrI) 0.71–0.98]. On subgroup analysis of individual drugs, there was no evidence of an association with viral suppression for 3TC (*n* = 184; HR 0.94; 95% CrI 0.73–1.15) or FTC (*n* = 454; HR 0.99; 95% CrI 0.80–1.19) amongst those on tenofovir‐containing regimens, but we estimated a reduced rate of viral suppression for people on 3TC amongst those without tenofovir use (*n* = 481; HR 0.71; 95% CrI 0.54–0.90). We found no association between 3TC/FTC and detection of any new DRM (overall HR 0.92; 95% CrI 0.64–1.18), but found inconclusive evidence of a lower incidence rate of new DRMs (overall incidence rate ratio 0.69; 95% CrI 0.34–1.11).

**Conclusions:**

We did not find evidence that 3TC or FTC use is associated with an increase in viral suppression, but it may reduce the appearance of additional DRMs in people with M184V/I. 3TC was associated with reduced viral suppression amongst people on regimens without tenofovir.

## Introduction

The HIV‐1 reverse transcriptase M184V/I mutation has historically been common in people living with HIV (PLHIV) experiencing virological failure on regimens that contain lamivudine (3TC) or emtricitabine (FTC). The mutation strongly reduces susceptibility to these drugs 1, but also leads to a reduction in viral fitness 2 and an increase in susceptibility to tenofovir (TFV), zidovudine and stavudine 3, 4. For most treatment decisions the presence of high‐level resistance to a particular drug would rule out its subsequent use. However, there has been a history of continuing 3TC/FTC in antiretroviral therapy (ART) regimens for some PLHIV with M184V/I because of the potential benefits of maintaining this mutation 5, 6 linked to impaired viral fitness and the finding that 3TC seems to retain some antiviral effect even in the presence of the M184V mutation 7.

The question of whether there is a benefit of maintaining 3TC/FTC in PLHIV with the M184V/I mutation remains relevant, particularly as there is current interest in dual‐therapy regimens (including some containing 3TC/FTC). One study found that boosted protease inhibitor (bPI) plus 3TC dual maintenance therapy for virally suppressed PLHIV with M184V at prior failure demonstrated an acceptably low failure rate at 48 weeks (3.0%), whereas bPI monotherapy did not (failure rate 24.8%) 8. This result reflects early ART trials that found zidovudine plus 3TC to be substantially more effective than zidovudine monotherapy 9, 10, despite the fact that high‐level resistance to 3TC develops rapidly without full viral suppression. 3TC monotherapy is also associated with better short‐term outcomes than complete treatment interruption amongst PLHIV with M184V 11.

One small randomized trial found that continuation of 3TC in PLHIV failing a 3TC‐containing regimen was not associated with any difference in change in viral load (VL) or CD4 cell count, but that continued presence of the M184V mutation was associated with a reduced rate of change of the viral sequence 12. There is also some *in vitro* evidence that M184V is associated with higher replication fidelity 13 and may delay or prevent the emergence of resistance to other ART drugs 14, 15. However, although there are some small case series 16, evidence is lacking for a protective effect of the M184V mutation from large cohort studies.

Through the analysis of UK HIV cohort data, we aimed to investigate whether 3TC/FTC use demonstrates any association with viral suppression or the occurrence of additional major drug resistance mutations (DRMs) following the detection of the M184V/I mutation.

## Methods

We considered all available samples in the UK HIV Drug Resistance Database (UK‐HDRD) obtained in the period 1997–2017. The prevalence of the M184V/I DRM was assessed in relation to calendar time stratified by whether PLHIV were recorded as ART‐naïve or ART‐experienced. Clinical data were obtained through linkage to the UK Collaborative HIV Cohort (UK CHIC) study 17.

For cases of M184V/I with clinical data available, we conducted time‐to‐event analyses for the outcomes of subsequent viral suppression and appearance of first new major DRM (i.e. mutations never detected prior to index ART switch) using International Antiviral Society–USA definitions 18. PLHIV were included in whom a change in ART regimen (or first‐line initiation) was recorded within 1 year of detection of M184V/I, with 3TC/FTC in the new regimen being the primary predictor of interest. The 1‐year cut‐off was chosen to ensure relevance of the index resistance test for the new ART regimen. Viral suppression was defined as a single viral load (VL) measurement  < 200 HIV‐1 RNA copies/mL; a single value was used because confirmatory VL measurements are not always available in retrospective data. New major DRMs were only included in analyses if they related to a drug class included in the switch regimen [e.g. if a patient were switched to a nucleoside reverse transcriptase inhibitor (NRTI) + protease inhibitor (PI) regimen, then a newly detected nonnucleoside reverse transcriptase inhibitor (NNRTI) resistance mutation would be ignored], and they were not counted if there was a historic DRM at the same codon.

Cox regression models were used, with follow‐up starting at the date of change to the ART regimen and with censoring at any further change to the ART regimen. For analysis of viral suppression, observations were also censored on the date of the last recorded VL on the index regimen, whilst for the analysis of any new DRM, observations were censored at the last VL or viral sequence only where no further ART change was recorded. People with viral suppression prior to ART regimen change after detection of the M184V/I mutation or in whom there were no VL measurements after ART change were not included. Time‐to‐event analyses for detection of any new DRM included all people meeting the criteria as for the analysis of viral suppression (those without any further viral sequences obtained were considered to be censored at the end of their follow‐up period with no event observed).

Due to the large number of distinct drug combinations used over the long time period analysed, the Cox models were stratified 19 by ART regimen considering all drugs other than 3TC/FTC (we term the regimen without considering 3TC/FTC use ‘ART_other_’). The effect of adding 3TC/FTC within ART_other_ group (i.e. conditional on the combination of other ART drugs) was analysed with adjustment for other individual and viral characteristics: baseline VL and CD4 count (latest within the 6‐month period prior to the start of the new ART regimen), age and calendar period at treatment change, ART‐naïve status, ethnicity, sex, exposure group, and number of reverse transcriptase and protease major DRMs present in the index viral sequence. The number of drugs with full or partial viral susceptibility (based on all prior viral sequences) was assessed using the Stanford HIVdb software (HIV Drug Resistance Database, Stanford University, Stanford, CA) and was also included.

We fitted Poisson models for the rate of appearance of all new DRMs, again stratified by ART_other_ regimen 20. Constant incidence of new DRMs within each person was assumed, and the follow‐up period for all people was defined as starting at the initial ART change and ending at the subsequent change to ART or the last VL measurement or resistance sequence where no further ART change was recorded. New DRMs were counted once per codon within the follow‐up period.

The initial analyses assumed a single constant hazard ratio (HR) or incidence rate ratio (IRR) for 3TC*/*FTC use across all ART_other_ strata for each outcome. However, we also carried out analyses generating separate effect estimates for 3TC *or* FTC use and according to whether the ART regimen contained TFV. The rationale for the latter subgroup division is the reported link between M184V/I and TFV sensitivity, and the fact that tenofovir disoproxil fumarate (TDF) became the most common choice of NRTI towards the end of the timeframe considered.

For Cox models, a random effect term (normally distributed on the log‐HR scale) was included grouped by the clinical centre that requested the index resistance sequence to allow for within‐centre correlation in outcomes. For Poisson models, person‐specific frailty terms were included to allow for differences in DRM incidence rate between people 21 along with centre‐specific frailty variables; these were log‐normally distributed (acting as an IRR). Regression models were fitted using a Bayesian approach with the rstan software 22, with posterior mean and 95% credibility intervals (CrIs) reported. Laplace priors were used for regression coefficients as described previously 23, with gamma (2,2) hyperpriors for the shrinkage parameter and for random effect scale parameters. Continuous predictor variables were transformed to a standardized scale for the regression [subtracting the mean and dividing by the standard deviation (SD)], and HRs and IRRs were estimated using mean‐centred linear spline functions with knots at −1, 0 and 1 (i.e. a relationship defined by a straight line with change of slope at three points).

## Results

A total of 9588 PLHIV had at least one viral sequence containing the M184V/I mutation in the UK‐HDRD in the time period considered. At first detection of M184V/I, 559 (5.8%) PLHIV were recorded as ART‐naïve and 8311 (86.7%) as ART‐experienced, with 718 (7.5%) without classification recorded. The prevalence of M184V/I in resistance tests of ART‐experienced PLHIV decreased substantially over the period 2002–2010, stabilizing at 10–15% beyond this (Fig. S1), whilst the prevalence in those recorded as ART‐naïve has been stable below 1% since 2006.

Linkage to any clinical data (in UK CHIC) was possible in 5068 PLHIV, with information on new ART regimen within 1 year of first detection of the M184V/I mutation in 3535 (excluding 32 people for whom the exact ART regimen was masked because of enrolment in a randomized trial).

Of the 3535 individuals in whom there was a change to the ART regimen (or ART initiation) within 1 year of detection of M184V/I: in 234 there was viral suppression prior to ART change, in 159 people the last recorded VL was prior to ART change and in 284 no VL measurements were recorded prior to another subsequent alteration of ART, resulting in 2858 people in whom virological response to ART switch could be evaluated. There were 17 people on 3TC/FTC monotherapy who were excluded from the analysis. Three further people were excluded for missing information on sex, four for missing age, and 237 for missing baseline CD4 count or VL prior to ART change.

A total of 2597 PLHIV were therefore included in the analyses for viral suppression and detection of new DRMs on a new ART regimen. Demographic and clinical characteristics of this group are summarized in Table 1. The median follow‐up time on the regimen started at index treatment change was 1.1 years [interquartile range (IQR) 0.4–2.9 years]. Overall, 3TC and/or FTC was used in 43.2% of these people, but there were strong trends over calendar time: 3TC use decreased from 56.1% in 1997 to < 20% from 2008, whilst FTC use increased from 0.6% in 2003 to 67.4% in 2017 (Table 2). From 2007 onwards, a majority had 3TC or FTC use.

**Table 1:** Demographic and clinical characteristics for 2597 people with antiretroviral therapy (ART) switch within 1 year of detection of the M184V/I mutation, according to use of lamivudine (3TC) or emtricitabine (FTC)

	Not on 3TC/FTC (*n* = 1474)	On 3TC/FTC (*n* = 1123)
Age at ART switch (years)	39 (34–45)	40 (34–45)
Date of ART switch	Sep 2002 (Jun 2000 to May 2006)	Oct 2006 (Mar 2001 to Mar 2011)
Baseline CD4 count (cells/μL)	240 (129–365)	260 (132–435)
Baseline VL (copies/mL)	13 000 (2800–68 600)	10 800 (1900–60 500)
Number of fully susceptible drugs		
None	134 (9)	184 (16)
One	423 (29)	304 (27)
Two	505 (34)	464 (41)
Three or more	412 (28)	171 (15)
Number of partially susceptible drugs		
None	748 (51)	642 (57)
One	501 (34)	382 (34)
Two	186 (13)	81 (7)
Three or more	39 (3)	18 (2)
Number of *pol* DRMs at baseline test		
One (M184V/I only)	194 (13)	215 (19)
Two	318 (22)	216 (19)
Three	280 (19)	188 (17)
Four	227 (15)	159 (14)
Five	153 (10)	110 (10)
Six or more	302 (20)	235 (21)
Exposure group		
MSM	741 (50)	566 (50)
Male MSW	251 (17)	206 (18)
Female WSM	308 (21)	244 (22)
Male IDU	28 (2)	24 (2)
Female IDU	17 (1)	10 (1)
Blood products	12 (0.8)	6 (0.5)
Mother to child	24 (2)	20 (2)
Other/unknown	93 (6)	47 (4)
Ethnicity		
White	776 (53)	574 (51)
Black Caribbean	46 (3)	30 (3)
Black African	427 (29)	372 (33)
Other/unknown	225 (15)	147 (13)
ART‐naïve	45 (3)	85 (8)

Data are shown as median (interquartile range) or *n* (%).

DRM, major drug resistance mutation; IDU, injecting drug user; MSM, men who have sex with men; MSW, men who have sex with women; VL, viral load; WSM, women who have sex with men.

**Table 2:** Lamivudine (3TC) or emtricitabine (FTC) use in people in whom change to antiretroviral therapy (ART) (or initiation if treatment‐naïve) was recorded within 1 year of first detection of the M184V/I mutation and who were recorded in the data set for analysis of time‐to‐event outcomes [requiring viral load (VL) follow‐up and baseline CD4 count and VL]

Year of ART change	On 3TC		On FTC		Not on 3TC/FTC		Total
	*n*	%	*n*	%	*n*	%	*n*
1997	37	56.1	0	0	29	43.9	66
1998	48	31.2	0	0	106	68.8	154
1999	86	35.8	0	0	154	64.2	240
2000	90	34.4	0	0	172	65.6	262
2001	64	26.9	0	0	173	73.0	237
2002	56	28.7	0	0	139	71.3	195
2003	50	32.3	1	0.6	104	67.1	155
2004	43	27.4	1	0.6	113	72.0	157
2005	32	24.4	13	9.9	86	65.6	131
2006	26	19.5	28	21.1	79	59.4	133
2007	33	25.2	38	29.0	60	45.8	131
2008	20	15.7	62	48.8	45	35.4	127
2009	11	11.2	51	52.0	36	36.7	98
2010	15	18.1	29	34.9	39	47.0	83
2011	11	13.4	40	48.8	31	37.8	82
2012	5	9.1	24	43.6	26	47.3	55
2013	13	15.7	43	51.8	27	32.5	83
2014	8	17.0	24	51.1	15	31.9	47
2015	4	6.7	37	61.7	19	31.7	60
2016	6	10.3	38	65.5	14	24.1	58
2017	7	16.3	29	67.4	7	16.3	43
Total	665	25.6	458	17.6	1474	56.8	2597

Of those people on FTC, nearly all were on tenofovir (TFV)‐containing regimens, the exceptions being two in 2005 and one each in 2009 and 2016.

Of the 2597 PLHIV included, only 89 were on a single ART drug without considering 3TC/FTC. Of these, 33 were on a boosted PI but in only three was this in conjunction with 3TC/FTC; none were on integrase inhibitor monotherapy or integrase inhibitor + 3TC/FTC dual therapy. There were 781 distinct ART regimens. Excluding 3TC/FTC from consideration, there were 616 distinct ART_other_ regimens (corresponding to strata in the models). If we consider those drug combinations (ART_other_) for which people were recorded in the data set both with and without 3TC/FTC use, there were 1684 people on 135 ART_other_ regimens. Only three patients in the sample were recorded as being on the tenofovir alafenamide (TAF) formulation of TFV, so this was considered interchangeable with TDF for the statistical analysis.

### Viral suppression after detection of M184V/I

Amongst the 2597 PLHIV included, overall virological suppression on the new ART regimen was 80% at 1 year and 83% at 2 years following switch (Kaplan–Meier estimates; Fig. S2). With adjustment for individual characteristics, we found a negative association between 3TC/FTC use and viral suppression < 200 copies/mL (HR 0.84; 95% CrI 0.71–0.98; Fig. S3).

**Figure 1:**
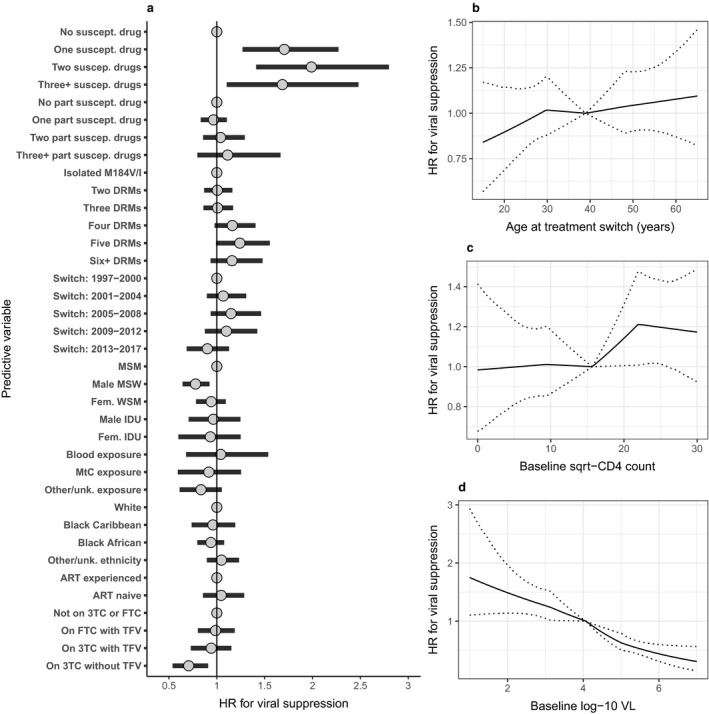
Associations between individual, viral and antiretroviral (ART) characteristics and viral suppression to < 200 copies/mL following ART switch subsequent to detection of the M184V/I mutation. Hazard ratios (HRs) were estimated through a Bayesian implementation of a Cox model, stratified by ART combination [± lamivudine/emtricitabine (3TC/FTC)] and with random effects for clinical centre. Categorical variables are shown in (a), with reference groups displayed as a fixed value of ‘1’. Associations between continuous variables of baseline (b) age, (c) CD4 count and (d) viral load (VL) and viral suppression are shown separately. Estimates are shown as posterior mean and 95% credibility interval. DRM, major drug resistance mutation; IDU, injecting drug user; MSM, men who have sex with men; MSW, men who have sex with women; MtC, mother‐to‐child; TFV, tenofovir; unk., unknown; WSM, women who have sex with men.

Generating separate effect estimates according to whether the regimen contained TFV and by 3TC or FTC use, we obtained results indicating no evidence of an association with viral suppression for those on 3TC (*n* = 184/1279; HR 0.94; 95% CrI 0.73–1.15) or FTC (*n* = 454/1279; HR 0.99; 95% CrI 0.80–1.19) amongst those on TFV‐containing regimens, but estimated a reduced rate of viral suppression for people on 3TC amongst those not on a TFV‐containing regimen (*n* = 481/1318; HR 0.71; 95% CrI 0.54–0.90) (Fig. 1). For this analysis, ‘not on 3TC or FTC’ was again the reference category for HRs, but different effects were estimated for FTC and for 3TC split by TFV use. Only four people were on FTC and non‐TFV regimens, so this combination could not be included. The differences observed were not altered by also considering interactions with other drugs for which the M184V/I mutation reduces (abacavir and didanosine) or increases (stavudine and zidovudine) susceptibility (Fig. S4). We also split non‐TFV regimens according to use of either stavudine or zidovudine and estimated a negative effect of 3TC/FTC use in both subgroups (Fig. S5).

Of the people on 3TC without TFV, 68% switched to the ART regimen for analysis prior to 2003. We therefore note that the evidence of a negative effect of 3TC use on virological suppression amongst people not on TFV‐containing regimens relates largely to ART regimens that have not been in use for many years; this observation was confirmed by comparison of the estimated effect of 3TC/FTC use ≤ 2006 (HR 0.82; 95% CrI 0.66–1.00) or ≥ 2007 (HR 0.91, 95% CrI 0.74–1.10) (Fig. S6).

For other individual characteristics in the model defined by TFV use, the strongest relationship with viral suppression was found for baseline VL, with HR ranging from 1.49 (95% CrI 1.14–1.97) for a low baseline VL of 100 copies/mL to 0.44 (95% CrI 0.31–0.60) for a high baseline VL of 1 000 000 copies/mL (relative to HR = 1 for mean of 11 900 copies/mL). The next strongest predictor of viral suppression was full viral susceptibility to one (HR 1.71; 95% CrI 1.27–2.27), two (HR 1.99; 95% CrI 1.40–2.80) or three or more (HR 1.69; 95% CrI 1.11–2.48) ART drugs within the regimen. High baseline CD4 count was associated with increased viral suppression (HR 1.21; 95% CrI 1.01–1.47 for 500 versus mean value of 245 cells/μL). Of the demographic characteristics considered, men who acquired HIV through sex with women showed evidence of reduced viral suppression (HR 0.78; 95% CrI 0.64–0.92) relative to men who acquired HIV through sex with men.

### Resistance mutations at follow‐up sequencing

**Figure 2:**
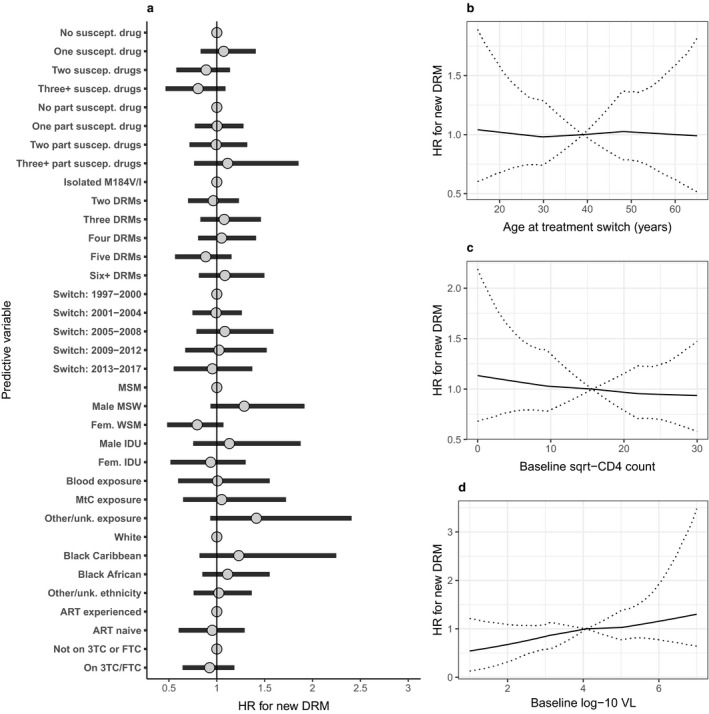
Associations between individual, viral and antiretroviral (ART) characteristics and detection of any new major drug resistance mutation (DRM) following ART switch subsequent to detection of the M184V/I mutation, with overall effect estimate for lamivudine (3TC) or emtricitabine (FTC) use. Hazard ratios (HRs) were estimated through a Bayesian implementation of a Cox model, stratified by ART combination (±3TC/FTC) and with random effects for clinical centre. Categorical variables are shown in (a), with reference groups displayed as a fixed value of ‘1’. Associations between continuous variables of baseline (b) age, (c) CD4 count and (d) viral load (VL) and detection of new DRMs are shown separately. Estimates are shown as posterior mean and 95% credibility interval. IDU, injecting drug user; MSM, men who have sex with men; MSW, men who have sex with women; MtC, mother‐to‐child; unk., unknown; WSM, women who have sex with men.

Of the 2597 PLHIV included, 698 had at least one resistance test whilst on the regimen started at the index treatment change, with 185 (26.5%) of these on 3TC and 102 (14.6%) on FTC. At least one new DRM was detected in 280 people (10.8%) during the follow‐up period considered. We did not find evidence that 3TC or FTC use was associated with reduced hazard of the first detection of a new DRM [overall HR 0.92; 95% CrI 0.64–1.18; *P*(HR < 1) = 0.71; Fig. 2]. When separate effect estimates were generated as for the analysis of viral suppression, we found no strong evidence of a relationship with new resistance for use of 3TC with TFV (new DRM in 23/184; HR 1.16; 95% CrI 0.80–1.93), FTC with TFV (new DRM in 17/454; HR 0.88; 95% CrI 0.45–1.23) or 3TC without TFV (new DRM in 54/481; HR 0.90; 95% CrI 0.57–1.18) (Fig. S7).

**Figure 3:**
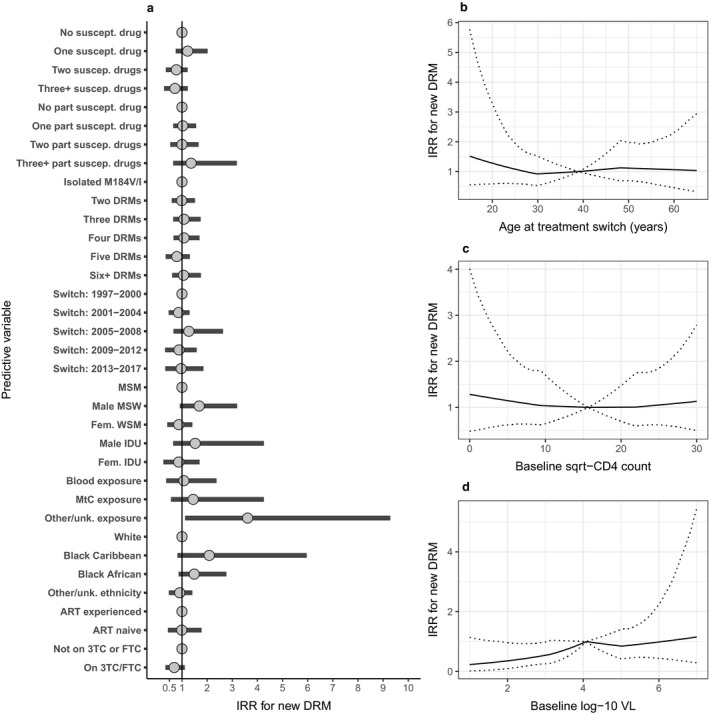
Associations between individual, viral and antiretroviral therapy (ART) characteristics and incidence rate of new viral major drug resistance mutations (DRMs) following ART switch subsequent to detection of the M184V/I mutation, with overall effect estimate for lamivudine (3TC) or emtricitabine (FTC). Incidence rate ratios (IRRs) were estimated through a Bayesian implementation of a Poisson model conditional on ART combination (±3TC/FTC) and with person‐specific frailty term and random effects for clinical centre. Categorical variables are shown in (a), with reference groups displayed as a fixed value of ‘1’. Associations between continuous variables of baseline (b) age, (c) CD4 count and (d) viral load (VL) and incidence of new DRMs are shown separately. Estimates are shown as posterior mean and 95% credibility interval. IDU, injecting drug user; MSM, men who have sex with men; MSW, men who have sex with women; MtC, mother‐to‐child; unk., unknown; WSM, women who have sex with men.

On analysis of the incidence of all new DRMs using a stratified Poisson model, we found inconclusive evidence of a reduction in incidence associated with 3TC/FTC use [IRR 0.69; 95% CrI 0.34–1.11; *P*(IRR < 1) = 0.92; Fig. 3]. We found no strong evidence of a relationship with DRM incidence for use of 3TC with TFV (IRR 1.11; 95% CrI 0.51–2.21), FTC with TFV (IRR 0.67; 95% CrI 0.18–1.23) or 3TC without TFV (IRR 0.79; 95% CrI 0.32–1.33) when considered separately (Fig. S8). We also split the effect of 3TC/FTC use by calendar period and found a stronger estimated reduction ≥ 2007 (IRR 0.62; 95% CrI 0.22–1.25) than ≤ 2006 (IRR 0.81; 95% CrI 0.41–1.34), although the result remained nondefinitive (Fig. S9).

For those people on 3TC/FTC, the M184V/I mutation remained in 59% of sequences obtained after ≥ 3 years of follow‐up (i.e. without further change to the ART regimen). However, for those not on 3TC/FTC, the proportion of sequences with the M184V/I mutation dropped from 18% 6 months to 1 year after ART regimen switch to 11% after ≥ 3 years (Table S1).

## Discussion

The use of 3TC or FTC was continued in 43.2% of PLHIV overall at ART switch following the detection of the M184V/I mutation, and from 2007 onwards one of these drugs was continued in the majority of people. We found evidence that use of 3TC was associated with reduced viral suppression amongst those on regimens without TFV, largely based on data now > 15 years old. However, we also found inconclusive evidence that the use of 3TC or FTC could be linked to a reduced incidence of new DRMs.

The high level of use of regimens containing FTC following the detection of M184V/I since 2007 can be attributed to the availability and widespread use of co‐formulated tablets with TDF [i.e. Truvada and Atripla (TDF/FTC/efavirenz)]. For PLHIV on regimens containing TFV and FTC, 99.3% (451/454) started the index regimen at a time when these drugs would have been available (following European Union licensing dates) in a combined tablet. For people on regimens containing TFV and 3TC, 63.0% (116/184) could have been taking 3TC in a combined tablet with another drug in their regimen. However, for people on 3TC on a regimen not containing TFV, at most 45.3% (218/481) could have been taking 3TC in a combined tablet. The observed negative association between 3TC use and viral suppression for those not on TFV might therefore be linked to higher pill burden 24. However, the observed association could also be attributable to uncontrolled confounding.

There was a large degree of variation in the ART regimens included in our analysis. This is a result of the timeframe considered, and the fact that regimens have been tailored to individuals based on their resistance tests. In order to address this issue, we carried out analyses with stratification for ART regimen, estimating the effect of adding 3TC or FTC to any given drug combination (assuming this to be constant). We focused on the potential added benefit of 3TC/FTC rather than evaluating the efficacy of specific ART regimens, which have improved greatly over time. The effect estimates calculated specifically for FTC relate to data from more recent calendar years, and so correspond to a more modern set of ART combinations than do those for 3TC.

Recent interest in the continued use of 3TC or FTC in the presence of the M184V/I mutation has focused on the topic of dual‐therapy regimens. This has been motivated by the desire to evaluate ART regimens with fewer drugs than the established standard of triple therapy including two NRTIs 25. 3TC is a particularly attractive choice for dual‐therapy ART as it is available as a low‐cost generic and has a well‐described favourable safety profile, and FTC is usually considered clinically equivalent despite some pharmacological differences 26.

There have been promising results for dual‐therapy regimens containing 3TC and either bPIs 27, 28, 29 or dolutegravir 30 both for first‐line ART and for maintenance therapy. The potential vulnerability of these regimens to compromise by the M184V/I mutation is a source of concern, as bPI 31 or dolutegravir 32 monotherapy is known to be suboptimal, although there is some evidence that bPI + 3TC dual maintenance therapy is effective in PLHIV with the previous detection of M184V 8, 33. Very few people in our data set were switched to monotherapy or dual therapy including 3TC or FTC following the detection of M184V/I, and so we were not able to evaluate 3TC/FTC use in this setting.

In our analysis of viral suppression following detection of M184V/I, we found that the most important factor in predicting success, other than baseline VL, was full susceptibility to at least one drug in the new regimen. There was no clear evidence of further improvement with full susceptibility to two or more drugs, or for the inclusion of drugs with partial susceptibility. This is consistent with secondary analyses of the SECOND‐LINE 34 and EARNEST 35 trials, which found that viral resistance to the NRTI backbone of second‐line bPI + NRTI regimens did not compromise virological outcomes. In the ODIN trial of darunavir‐based ART, the presence of the M184V/I mutation at baseline was predictive of successful viral suppression 36.

Although there is no definitive evidence, it is widely thought that the positive correlation between baseline DRMs and the success of second‐line therapy 34, 35 may reflect suboptimal ART adherence in those PLHIV who fail first‐line treatment without resistance 37, 38. Another possible explanation for the effectiveness of regimens that include NRTIs with limited predicted viral susceptibility is the impact of the mutations on viral fitness following introduction of a new antiretroviral agent; this was raised by the authors of another secondary analysis of a second‐line bPI + NRTI trial that found that virological response was not affected by NRTI resistance 39. We included the total number of DRMs prior to ART switch in our analyses to evaluate whether accumulation of DRMs is associated with viral suppression, conditional on effectiveness of the new regimen, but did not find strong evidence of a relationship.

We did not find that continued 3TC or FTC use was associated with reduced risk of first detection of new DRMs following ART switch in PLHIV with M184V/I, but we did find some evidence for a reduced incidence rate of new DRMs over the entire follow‐up period. Although these results taken together are not definitive, the analysis carried out was Bayesian and the credibility intervals obtained can therefore be interpreted in a directly probabilistic manner. Although not proven, a reduction in the incidence of new DRMs would be consistent with increased HIV replication fidelity 13 linked to maintenance of the M184V mutation; we confirmed that the M184V/I mutation could be detected in a majority of the available follow‐up sequences among patients on 3TC/FTC, but was absent in > 80% of follow‐up sequences > 6 months from switch for those people not on 3TC/FTC. One *in vitro* study found that the presence of the M184V/I mutation prevented the appearance of DRMs for HIV‐infected tissue cultures exposed to dolutegravir, but not for those exposed to raltegravir or elvitegravir 15. Earlier tissue culture studies found that the M184V/I mutation delayed emergence of resistance to the NNRTI efavirenz and to the PI amprenavir 14, although no such effect was found for nevirapine 14, 40 or ritonavir 41. It is therefore possible that maintenance of M184V/I is beneficial in this regard for some ART regimens but not others.

We did not find evidence of a benefit of 3TC or FTC use following the detection of the M184V/I mutation in terms of viral suppression in our retrospective analysis of routine clinical data. However, our results do provide some limited evidence that use of 3TC or FTC may help to reduce the incidence of additional DRMs. Where randomized or other high‐quality evidence exists for specific ART regimens, this should be used to guide judgements regarding the use of 3TC or FTC in PLHIV with the M184V/I mutation present.

## Supporting information


**Table S1** Presence of the M184V/I mutation following antiretroviral therapy (ART) switch subsequent to initial detection of the M184V/I mutation
**Fig. S1** Prevalence of the M184V/I mutation per person living with HIV (PLHIV) by calendar year of sequencing (people can be included in multiple calendar years, but are only counted once per year), according to whether the person was antiretroviral therapy (ART)‐experienced (black circle) or naïve (orange circle) at the time of blood sample.
**Fig. S2** Kaplan–Meier plot of virological suppression (to < 200 copies/mL) amongst the 2597 people included in the time‐to‐event analyses. The 95% confidence interval is shown by shaded area.
**Fig. S3** Associations between individual, viral and antiretroviral therapy (ART) characteristics and viral suppression to < 200 copies/mL following ART switch subsequent to detection of the M184V/I mutation, with overall effect estimate for lamivudine (3TC) or emtricitabine (FTC) use. Hazard ratios (HRs) were estimated through a Bayesian implementation of a Cox model, stratified by ART combination (±3TC/FTC) and with random effects for clinical centre. Categorical variables are shown in (a), with reference groups displayed as a fixed value of ‘1’. Associations between continuous variables of baseline (b) age, (c) CD4 count and (d) VL and viral suppression are shown separately. Estimates are shown as posterior mean and 95% credibility interval.
**Fig. S4** Associations between individual, viral and antiretroviral therapy (ART) characteristics and viral suppression to < 200 copies/mL following ART switch subsequent to detection of the M184V/I mutation, with interactions for abacavir (ABC), didanosine (DDI), stavudine (D4T) and zidovudine (ZDV) use. Hazard ratios (HRs) were estimated through a Bayesian implementation of a Cox model, stratified by ART combination (±3TC/FTC) and with random effects for clinical centre. Categorical variables are shown in (a), with reference groups displayed as a fixed value of ‘1’. Associations between continuous variables of baseline (b) age, (c) CD4 count and (d) viral load (VL) and viral suppression are shown separately. Estimates are shown as posterior mean and 95% credibility interval.
**Fig. S5** Associations between individual, viral and antiretroviral therapy (ART) characteristics and viral suppression to < 200 copies/mL following ART switch subsequent to detection of the M184V/I mutation, with effect of lamivudine/emtricitabine (3TC/FTC) separated according to use of either tenofovir (TFV) or zidovudine (ZDV)/stavudine (D4T) without TFV. Hazard ratios (HRs) were estimated through a Bayesian implementation of a Cox model, stratified by ART combination (±3TC/FTC) and with random effects for clinical centre. Categorical variables are shown in (a), with reference groups displayed as a fixed value of ‘1’. Associations between continuous variables of baseline (b) age, (c) CD4 count and (d) viral load (VL) and viral suppression are shown separately. Estimates are shown as posterior mean and 95% credibility interval.
**Fig. S6** Associations between individual, viral and antiretroviral therapy (ART) characteristics and viral suppression to < 200 copies/mL following ART switch subsequent to detection of the M184V/I mutation, with separate effect estimates for lamivudine (3TC) or emtricitabine (FTC) use before or after 2007. Hazard ratios (HRs) were estimated through a Bayesian implementation of a Cox model, stratified by ART combination (±3TC/FTC) and with random effects for clinical centre. Categorical variables are shown in (a), with reference groups displayed as a fixed value of ‘1’. Associations between continuous variables of baseline (b) age, (c) CD4 count and (d) viral load (VL) and viral suppression are shown separately. Estimates are shown as posterior mean and 95% credibility interval.
**Fig. S7** Associations between individual, viral and antiretroviral therapy (ART) characteristics and detection of any new viral drug resistance mutation (DRM) following ART switch subsequent to detection of the M184V/I mutation. Hazard ratios (HRs) were estimated through a Bayesian implementation of a Cox model, stratified by ART combination (±3TC/FTC) and with random effects for clinical centre. Categorical variables are shown in (a), with reference groups displayed as a fixed value of ‘1’. Associations between continuous variables of baseline (b) age, (c) CD4 count and (d) viral load (VL) and detection of new DRMs are shown separately. Estimates are shown as posterior mean and 95% credibility interval.
**Fig. S8** Associations between individual, viral and antiretroviral therapy (ART) characteristics and incidence rate of new viral drug resistance mutations (DRMs) following ART switch subsequent to detection of the M184V/I mutation. Incidence rate ratios (IRRs) were estimated through a Bayesian implementation of a Poisson model conditional on ART combination (±3TC/FTC) and with person‐specific frailty term and random effects for clinical centre. Categorical variables are shown in (a), with reference groups displayed as a fixed value of ‘1’. Associations between continuous variables of baseline (b) age, (c) CD4 count and (d) viral load (VL) and incidence of new DRMs are shown separately. Estimates are shown as posterior mean and 95% credibility interval.
**Fig. S9** Associations between individual, viral and antiretroviral therapy (ART) characteristics and incidence rate of new viral drug resistance mutations (DRMs) following ART switch subsequent to detection of the M184V/I mutation, with separate effect estimates for lamivudine (3TC) or emtricitabine (FTC) use before or after 2007. Incidence rate ratios (IRRs) were estimated through a Bayesian implementation of a Poisson model conditional on ART combination (±3TC/FTC) and with person‐specific frailty term and random effects for clinical centre. Categorical variables are shown in (a), with reference groups displayed as a fixed value of ‘1’. Associations between continuous variables of baseline (b) age, (c) CD4 count and (d) viral load (VL) and incidence of new DRMs are shown separately. Estimates are shown as posterior mean and 95% credibility interval.

## Appendix 1

### UK HIV Drug Resistance Database

**Steering committee:** David Asboe, Anton Pozniak (Chelsea & Westminster Hospital, London); Patricia Cane (Public Health England, Porton Down); David Chadwick (South Tees Hospitals NHS Trust, Middlesbrough); Duncan Churchill (Brighton and Sussex University Hospitals NHS Trust, Brighton); Duncan Clark (Barts Health NHS Trust, London); Simon Collins (HIV i‐Base, London); Valerie Delpech (National Infection Service, Public Health England); Samuel Douthwaite (Guy’s and St. Thomas’ NHS Foundation Trust, London); David Dunn, Esther Fearnhill, Kholoud Porter, Anna Tostevin, Oliver Stirrup (Institute for Global Health, UCL, London); Christophe Fraser (University of Oxford,Oxford); Anna Maria Geretti (Institute of Infection and Global Health, University of Liverpool, Liverpool); Rory Gunson (Gartnavel General Hospital, Glasgow); Antony Hale (Leeds Teaching Hospitals NHS Trust, Leeds); Stéphane Hué (London School of Hygiene and Tropical Medicine, London); Linda Lazarus (Expert Advisory Group on AIDS Secretariat, Public Health England); Andrew Leigh‐Brown (University of Edinburgh, Edinburgh); Tamyo Mbisa (National Infection Service, Public Health England); Nicola Mackie (Imperial NHS Trust, London); Chloe Orkin (Barts Health NHS Trust, London); Eleni Nastouli, Deenan Pillay, Andrew Phillips, Caroline Sabin (University College London, London); Erasmus Smit (Public Health England, Birmingham Heartlands Hospital, Birmingham); Kate Templeton (Royal Infirmary of Edinburgh, Edinburgh); Peter Tilston (Manchester Royal Infirmary, Manchester); Erik Volz (Imperial College London, London); Ian Williams (Mortimer Market Centre, London); Hongyi Zhang (Addenbrooke’s Hospital, Cambridge).

**Coordinating centre:** Institute for Global Health, UCL, London (David Dunn, Keith Fairbrother, Esther Fearnhill, Kholoud Porter, Anna Tostevin, Oliver Stirrup).

**Centres contributing data:** Clinical Microbiology and Public Health Laboratory, Addenbrooke’s Hospital, Cambridge (Justine Dawkins); Guy’s and St Thomas’ NHS Foundation Trust, London (Siobhan O’Shea, Jane Mullen); PHE – Public Health Laboratory, Birmingham Heartlands Hospital, Birmingham (Erasmus Smit); Antiviral Unit, National Infection Service, Public Health England, London (Tamyo Mbisa); Imperial College Health NHS Trust, London (Alison Cox); King’s College Hospital, London (Richard Tandy); Medical Microbiology Laboratory, Leeds Teaching Hospitals NHS Trust, Leeds (Tracy Fawcett); Specialist Virology Centre, Liverpool (Mark Hopkins); Department of Clinical Virology, Manchester Royal Infirmary, Manchester (Peter Tilston); Department of Virology, Royal Free Hospital, London (Clare Booth, Ana Garcia‐Diaz); Edinburgh Specialist Virology Centre, Royal Infirmary of Edinburgh, Edinburgh (Lynne Renwick); Department of Infection & Tropical Medicine, Royal Victoria Infirmary, Newcastle (Matthias L. Schmid, Brendan Payne); South Tees Hospitals NHS Trust, Middlesbrough (David Chadwick); Department of Virology, Barts Health NHS Trust, London (Jonathan Hubb); Molecular Diagnostic Unit, Imperial College, London (Simon Dustan); University College London Hospitals, London (Stuart Kirk); West of Scotland Specialist Virology Laboratory, Gartnavel, Glasgow (Rory Gunson, Amanda Bradley‐Stewart).

### UK Collaborative HIV Cohort

**Steering committee:** Jonathan Ainsworth, Sris Allan, Jane Anderson, Ade Apoola, David Chadwick, Duncan Churchill, Valerie Delpech, David Dunn, Ian Fairley, Ashini Fox, Richard Gilson, Mark Gompels, Phillip Hay, Rajesh Hembrom, Teresa Hill, Margaret Johnson, Sophie Jose, Stephen Kegg, Clifford Leen, Dushyant Mital, Mark Nelson, Hajra Okhai, Chloe Orkin, Adrian Palfreeman, Andrew Phillips, Deenan Pillay, Ashley Price, Frank Post, Jillian Pritchard, Caroline Sabin, Achim Schwenk, Anjum Tariq, Roy Trevelion, Andy Ustianowski, John Walsh.

**Central co‐ordination**: University College London (David Dunn, Teresa Hill, Hajra Okhai, Andrew Phillips, Caroline Sabin); Medical Research Council Clinical Trials Unit at UCL (MRC CTU at UCL), London (Nadine van Looy, Keith Fairbrother).

**Participating centres:** Barts Health NHS Trust, London (Chloe Orkin, Janet Lynch, James Hand); Brighton and Sussex University Hospitals NHS Trust, Brighton (Duncan Churchill, Stuart Tilbury, Elaney Youssef, Duncan Churchill); Chelsea and Westminster Hospital NHS Foundation Trust, London (Mark Nelson, Richard Daly, David Asboe, Sundhiya Mandalia); Homerton University Hospital NHS Trust, London (Jane Anderson, Sajid Munshi); King’s College Hospital NHS Foundation Trust, London (Frank Post, Ade Adefisan, Chris Taylor, Zachary Gleisner, Fowzia Ibrahim, Lucy Campbell); South Tees Hospitals NHS Foundation Trust, Middlesbrough (David Chadwick, Kirsty Baillie); Mortimer Market Centre, University College London, London (Richard Gilson, Ian Williams); North Middlesex University Hospital NHS Trust, London (Jonathan Ainsworth, Achim Schwenk, Sheila Miller, Chris Wood); Royal Free NHS Foundation Trust/University College London, London (Margaret Johnson, Mike Youle, Fiona Lampe, Colette Smith, Rob Tsintas, Clinton Chaloner, Caroline Sabin, Andrew Phillips, Teresa Hill, Hajra Okhai); Imperial College Healthcare NHS Trust, London (John Walsh, Nicky Mackie, Alan Winston, Jonathan Weber, Farhan Ramzan, Mark Carder); The Lothian University Hospitals NHS Trust, Edinburgh (Clifford Leen, Andrew Kerr, David Wilks, Sheila Morris); North Bristol NHS Trust, Bristol (Mark Gompels, Sue Allan); University Hospitals of Leicester NHS Trust, Leicester (Adrian Palfreeman, Adam Lewszuk); Woolwich, Lewisham and Greenwich NHS Trust, London (Stephen Kegg, Victoria Ogunbiyi, Sue Mitchell), St. George’s Healthcare NHS Trust, London (Phillip Hay, Christopher Hunt, Olanike Okolo, Benjamin Watts); York Teaching Hospital NHS Foundation Trust, York (Ian Fairley, Sarah Russell‐Sharpe, Olatunde Fagbayimu); University Hospitals Coventry and Warwickshire NHS Trust, Coventry (Sris Allan, Debra Brain); The Royal Wolverhampton Hospitals NHS Trust, Wolverhampton (Anjum Tariq, Liz Radford, Sarah Milgate); Chertsey, Ashford and St. Peter’s Hospitals NHS Foundation Trust (Jillian Pritchard, Shirley Cumming, Claire Atkinson); Milton Keynes Hospital NHS Foundation Trust, Milton Keynes (Dushyant Mital, Annie Rose, Jeanette Smith); The Pennine Acute Hospitals NHS Trust (Andy Ustianowski, Cynthia Murphy, Ilise Gunder); Nottingham University Hospitals NHS Trust, Nottingham (Ashini Fox, Howard Gees, Gemma Squires, Laura Anderson), Kent Community Health NHS Foundation Trust (Rajesh Hembrom, Serena Mansfield, Lee Tomlinson, Christine LeHegerat, Roberta Box, Tom Hatton, Doreen Herbert), The Newcastle upon Tyne Hospitals NHS Foundation Trust, Newcastle (Ashley Price, Ian McVittie, Victoria Murtha, Laura Shewan); Derby Teaching Hospitals NHS Foundation Trust, Derby (Ade Apoola, Zak Connan, Luke Gregory, Kathleen Holding, Victoria Chester, Trusha Mistry, Catherine Gatford); Public Health England, London (Valerie Delpech); i‐Base (Roy Trevelion).
