# Supplementary material for: Continuation of emtricitabine/lamivudine within combination antiretroviral therapy following detection of the M184V/I HIV‐1 resistance mutation
Source: HIV Med. 2020 Jan 11;21(5):309–21. doi: 10.1111/hiv.12829 (PMC7217157; doi:10.1111/hiv.12829)
Supplement: Supplementary file 1 — Table S1 Presence of the M184V/I mutation following antiretroviral therapy (ART) switch subsequent to initial detection of the M184V/I mutation Fig. S1 Prevalence of the M184V/I mutation per person living with HIV (PLHIV) by calendar year of sequencing (people can be included in multiple calendar years, but are only counted once per year), according to whether the person was antiretroviral therapy (ART)‐experienced (black circle) or naïve (orange circle) at the time of blood sample. Fig. S2 Kaplan–Meier plot of virological suppression (to < 200 copies/mL) amongst the 2597 people included in the time‐to‐event analyses. The 95% confidence interval is shown by shaded area. Fig. S3 Associations between individual, viral and antiretroviral therapy (ART) characteristics and viral suppression to < 200 copies/mL following ART switch subsequent to detection of the M184V/I mutation, with overall effect estimate for lamivudine (3TC) or emtricitabine (FTC) use. Hazard ratios (HRs) were estimated through a Bayesian implementation of a Cox model, stratified by ART combination (±3TC/FTC) and with random effects for clinical centre. Categorical variables are shown in (a), with reference groups displayed as a fixed value of ‘1’. Associations between continuous variables of baseline (b) age, (c) CD4 count and (d) VL and viral suppression are shown separately. Estimates are shown as posterior mean and 95% credibility interval. Fig. S4 Associations between individual, viral and antiretroviral therapy (ART) characteristics and viral suppression to < 200 copies/mL following ART switch subsequent to detection of the M184V/I mutation, with interactions for abacavir (ABC), didanosine (DDI), stavudine (D4T) and zidovudine (ZDV) use. Hazard ratios (HRs) were estimated through a Bayesian implementation of a Cox model, stratified by ART combination (±3TC/FTC) and with random effects for clinical centre. Categorical variables are shown in (a), with reference groups displayed as a fixed value of [file HIV-21-309-s001.docx]

**Table S1** Presence of M184V/I mutation following ART switch subsequent to initial detection of the M184V/I mutation. Results are reported on a per sequence basis, with sequences only included for people ‘in follow-up’ for the time-to-event analyses (i.e. without further change to ART regimen).

| Time sequence obtained from ART switch | Person not on 3TC/FTC | | Person on 3TC/FTC | |
| --- | --- | --- | --- | --- |
|  | *n* | *n with M184V/I (%)* | *n* | *n with M184V/I (%)* |
| 0–6 months | 141 | 63 (45) | 132 | 90 (68) |
| 6 months –1 year | 143 | 26 (18) | 103 | 66 (64) |
| 1–2 years | 145 | 20 (14) | 95 | 52 (55) |
| 2–3 years | 78 | 10 (13) | 71 | 39 (55) |
| 3+ years | 135 | 15 (11) | 99 | 58 (59) |

3TC, lamivudine; ART, antiretroviral therapy; FTC, emtricitabine.

**Figure S1** Prevalence of the M184V/I mutation per person living with HIV (PLHIV) by calendar year of sequencing (people can be included in multiple calendar years, but are only counted once per year), according to whether the person was ART experienced (black circle) or naïve (orange circle) at the time of blood sample. The denominator in each year is the total number of PLHIV with at least one reverse transcriptase sequence recorded in that year. Binomial 95% CIs are shown


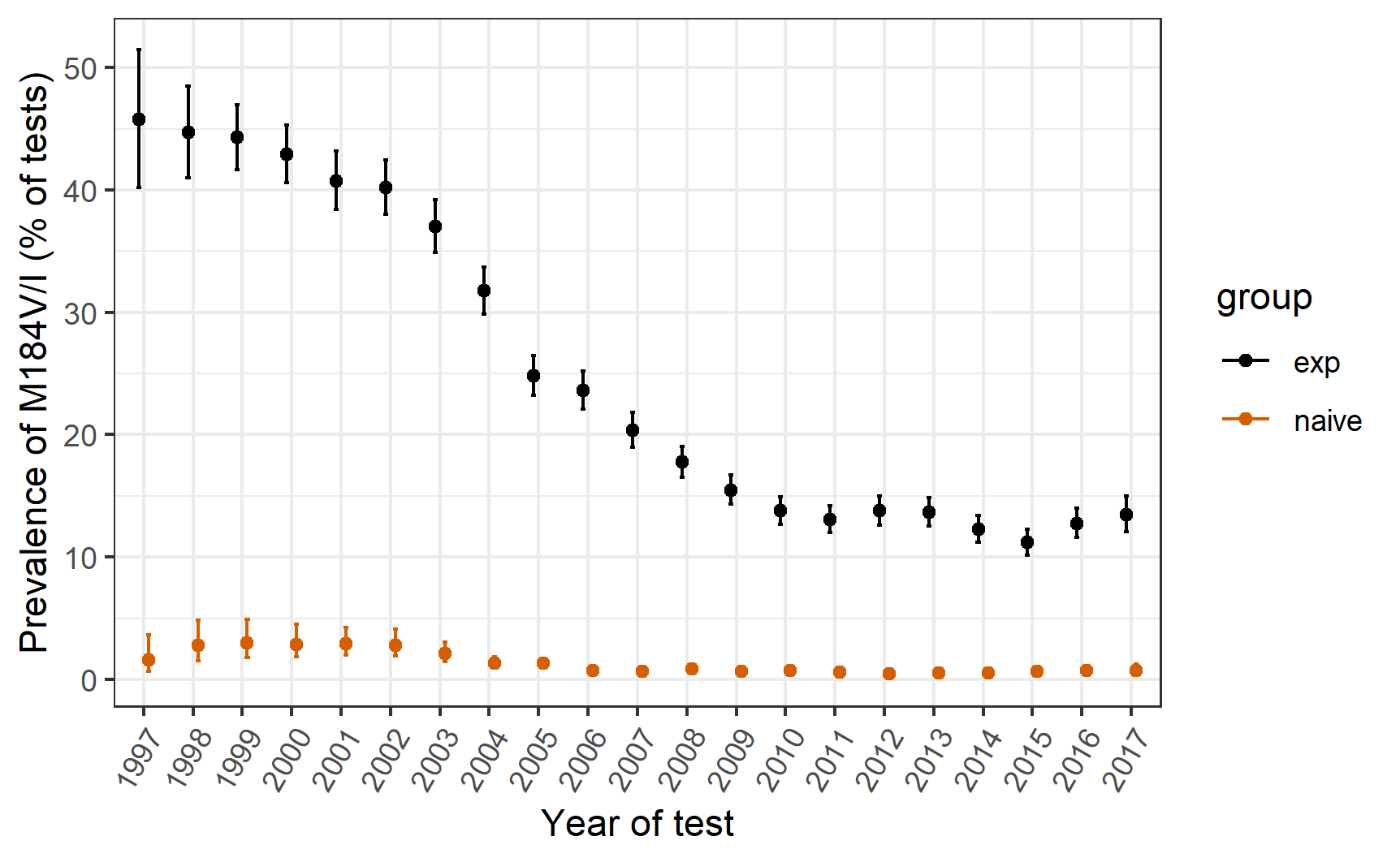


People living with HIV without classification recorded were excluded for the evaluation of prevalence, but ART-status was available in all UK CHIC-linked individuals for the analyses of subsequent viral suppression and drug resistance outcomes. ART, antiretroviral therapy.

**Figure S2** Kaplan–Meier plot of virological suppression (to <200 copies/mL) amongst the 2597 people included in the time-to-event analyses. 95% CI is shown by shaded area.

**Figure S3** Associations between individual, viral and ART characteristics and viral suppression to <200 copies/mL following ART switch subsequent to detection of the M184V/I mutation, with overall effect estimate for 3TC or FTC use. Hazard ratios (HR) estimated through a Bayesian implementation of a Cox model, stratified by ART combination (±3TC/FTC) and with random effects for clinical centre. Categorical variables are shown in (a), with reference groups displayed as a fixed value of ‘1’. Associations between continuous variables of baseline (b) age, (c) CD4 count and (d) VL and viral suppression are shown separately. Estimates are shown as posterior mean and 95% credibility interval.


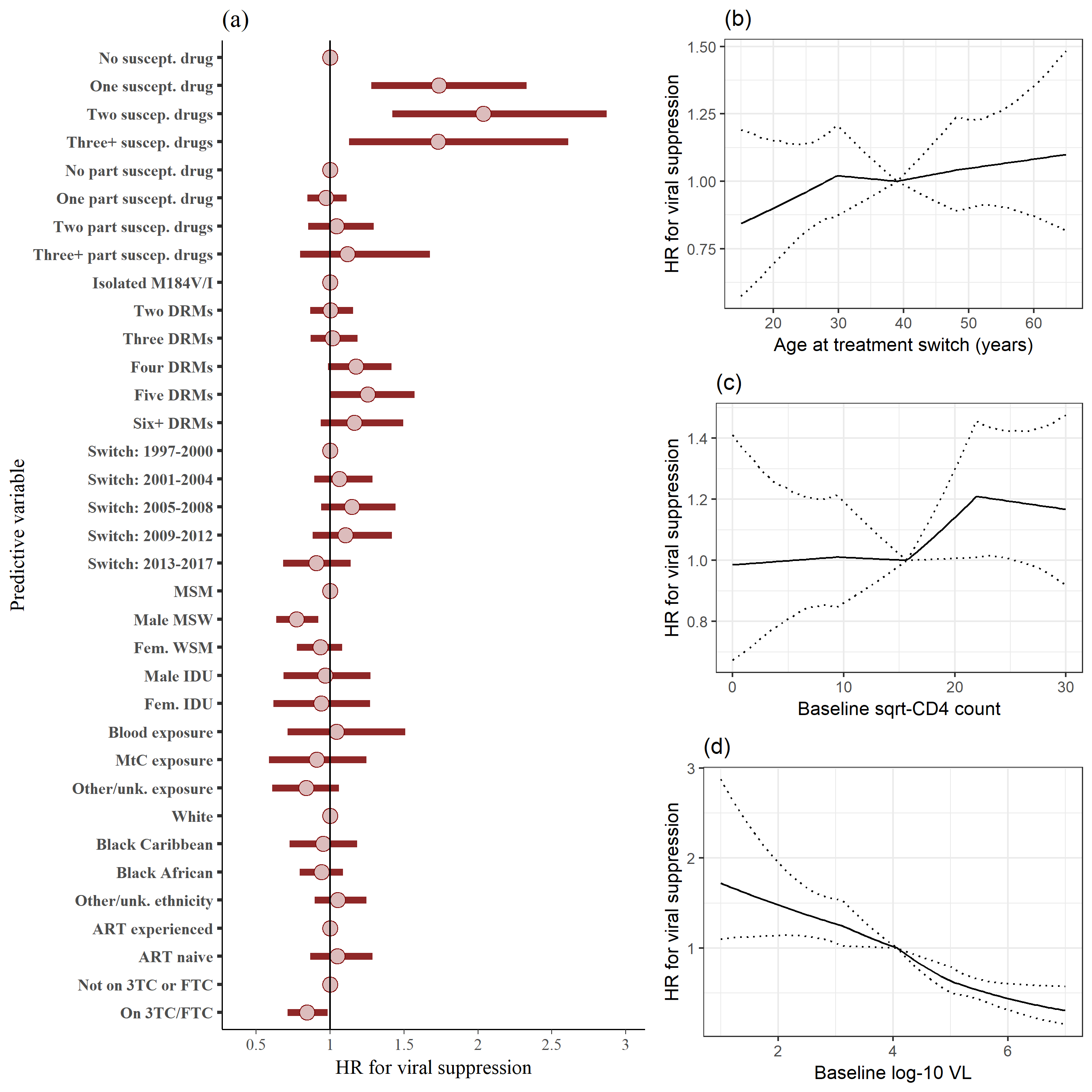


3TC, lamivudine; ART, antiretroviral therapy; DRM, major drug resistance mutation; FTC, emtricitabine; IDU, injecting drug user; MSM, men who have sex with men; MSW, men who have sex with women; MtC, mother-to child; unk., unknown; VL, viral load; WSM, women who have sex with men.

**Figure S4** Associations between individual, viral and ART characteristics and viral suppression to <200 copies/mL following ART switch subsequent to detection of the M184V/I mutation, with interactions for abacavir (ABC), didanosine (DDI), stavudine (D4T) and zidovudine (ZDV) use. Hazard ratios (HR) estimated through a Bayesian implementation of a Cox model, stratified by ART combination (±3TC/FTC) and with random effects for clinical centre. Categorical variables are shown in (a), with reference groups displayed as a fixed value of ‘1’. Associations between continuous variables of baseline (b) age, (c) CD4 count and (d) VL and viral suppression are shown separately. Estimates are shown as posterior mean and 95% credibility interval.


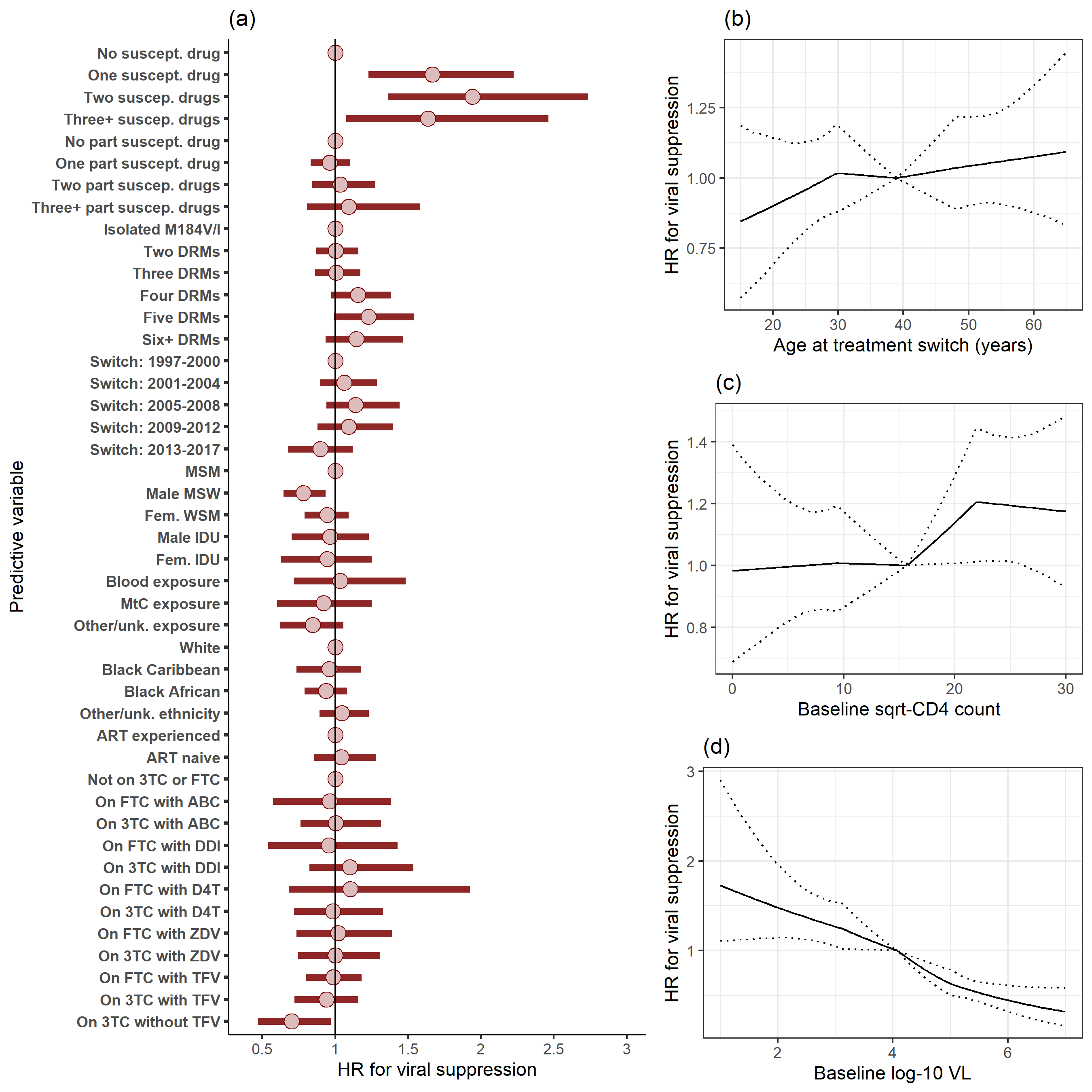


3TC, lamivudine; ART, antiretroviral therapy; DRM, major drug resistance mutation; FTC, emtricitabine; IDU, injecting drug user; MSM, men who have sex with men; MSW, men who have sex with women; MtC, mother-to child; TFV, tenofovir; unk., unknown; VL, viral load; WSM, women who have sex with men.

**Figure S5** Associations between individual, viral and ART characteristics and viral suppression to <200 copies/mL following ART switch subsequent to detection of the M184V/I mutation, with effect of 3TC/FTC separated according to use of either tenofovir (TFV) or zidovudine (ZDV)/stavudine (D4T) without TFV. Hazard ratios (HR) estimated through a Bayesian implementation of a Cox model, stratified by ART combination (±3TC/FTC) and with random effects for clinical centre. Categorical variables are shown in (a), with reference groups displayed as a fixed value of ‘1’. Associations between continuous variables of baseline (b) age, (c) CD4 count and (d) VL and viral suppression are shown separately. Estimates are shown as posterior mean and 95% credibility interval.


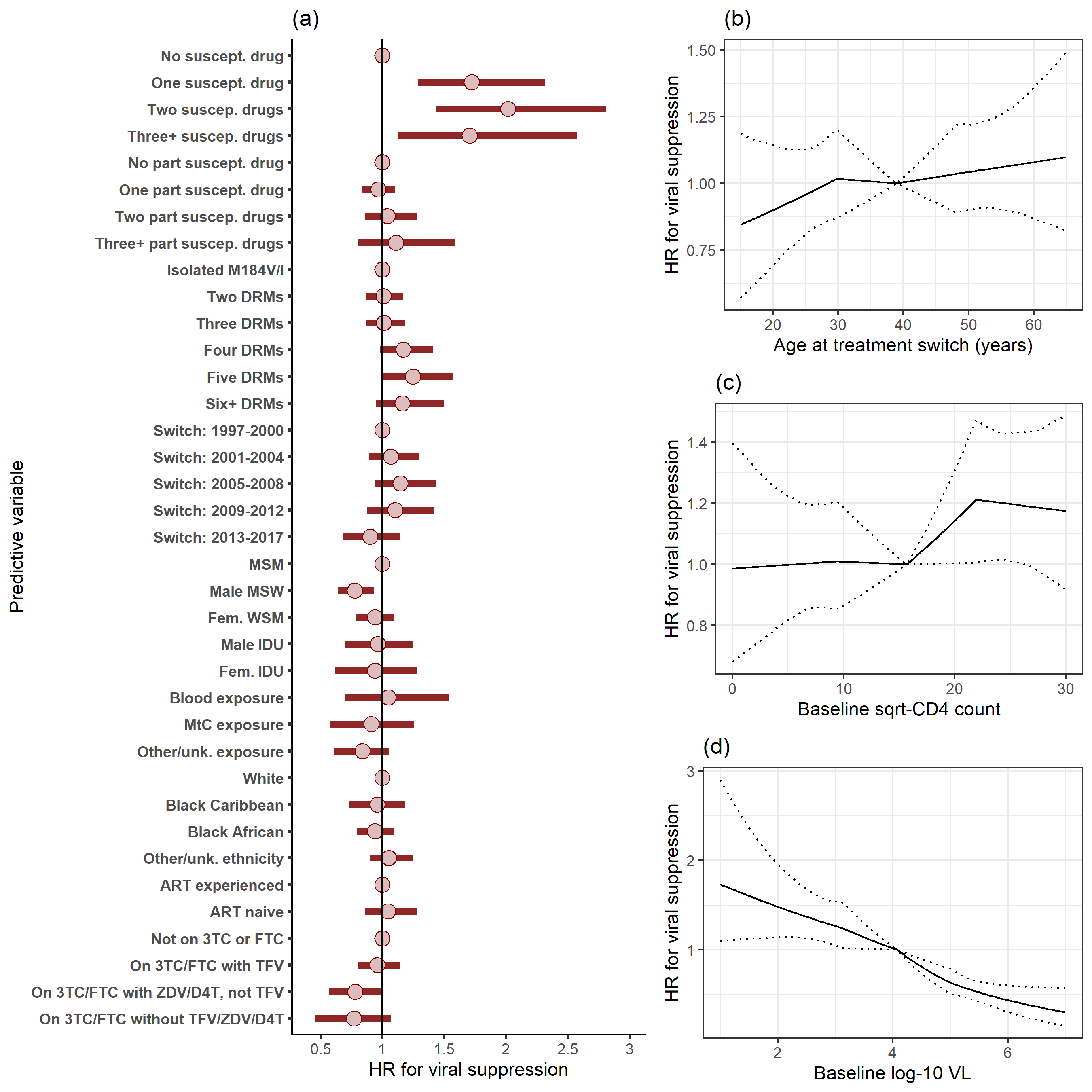


3TC, lamivudine; ART, antiretroviral therapy; DRM, major drug resistance mutation; FTC, emtricitabine; IDU, injecting drug user; MSM, men who have sex with men; MSW, men who have sex with women; MtC, mother-to child; TFV, tenofovir; unk., unknown; VL, viral load; WSM, women who have sex with men.

**Figure S6** Associations between individual, viral and ART characteristics and viral suppression to <200 copies/mL following ART switch subsequent to detection of the M184V/I mutation, with separate effect estimates for 3TC or FTC use before or after 2007. Hazard ratios (HR) estimated through a Bayesian implementation of a Cox model, stratified by ART combination (±3TC/FTC) and with random effects for clinical centre. Categorical variables are shown in (a), with reference groups displayed as a fixed value of ‘1’. Associations between continuous variables of baseline (b) age, (c) CD4 count and (d) VL and viral suppression are shown separately. Estimates are shown as posterior mean and 95% credibility interval.


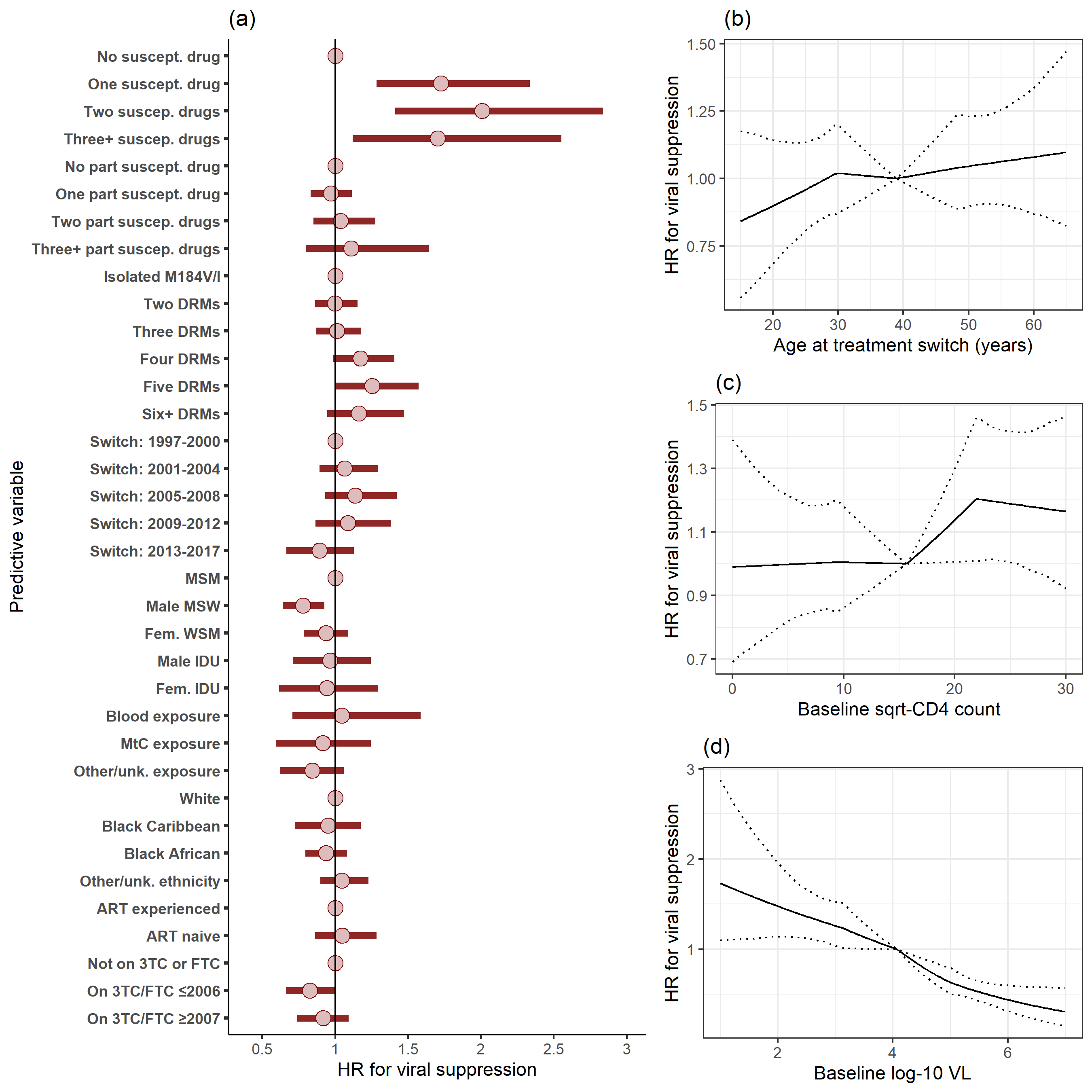


3TC, lamivudine; ART, antiretroviral therapy; DRM, major drug resistance mutation; FTC, emtricitabine; IDU, injecting drug user; MSM, men who have sex with men; MSW, men who have sex with women; MtC, mother-to child; unk., unknown; VL, viral load; WSM, women who have sex with men.

**Figure S7** Associations between individual, viral and ART characteristics and detection of any new viral DRM following ART switch subsequent to detection of the M184V/I mutation. Hazard ratios (HR) estimated through a Bayesian implementation of a Cox model, stratified by ART combination (±3TC/FTC) and with random effects for clinical centre. Categorical variables are shown in (a), with reference groups displayed as a fixed value of ‘1’. Associations between continuous variables of baseline (b) age, (c) CD4 count and (d) VL and detection of new DRM are shown separately. Estimates are shown as posterior mean and 95% credibility interval.


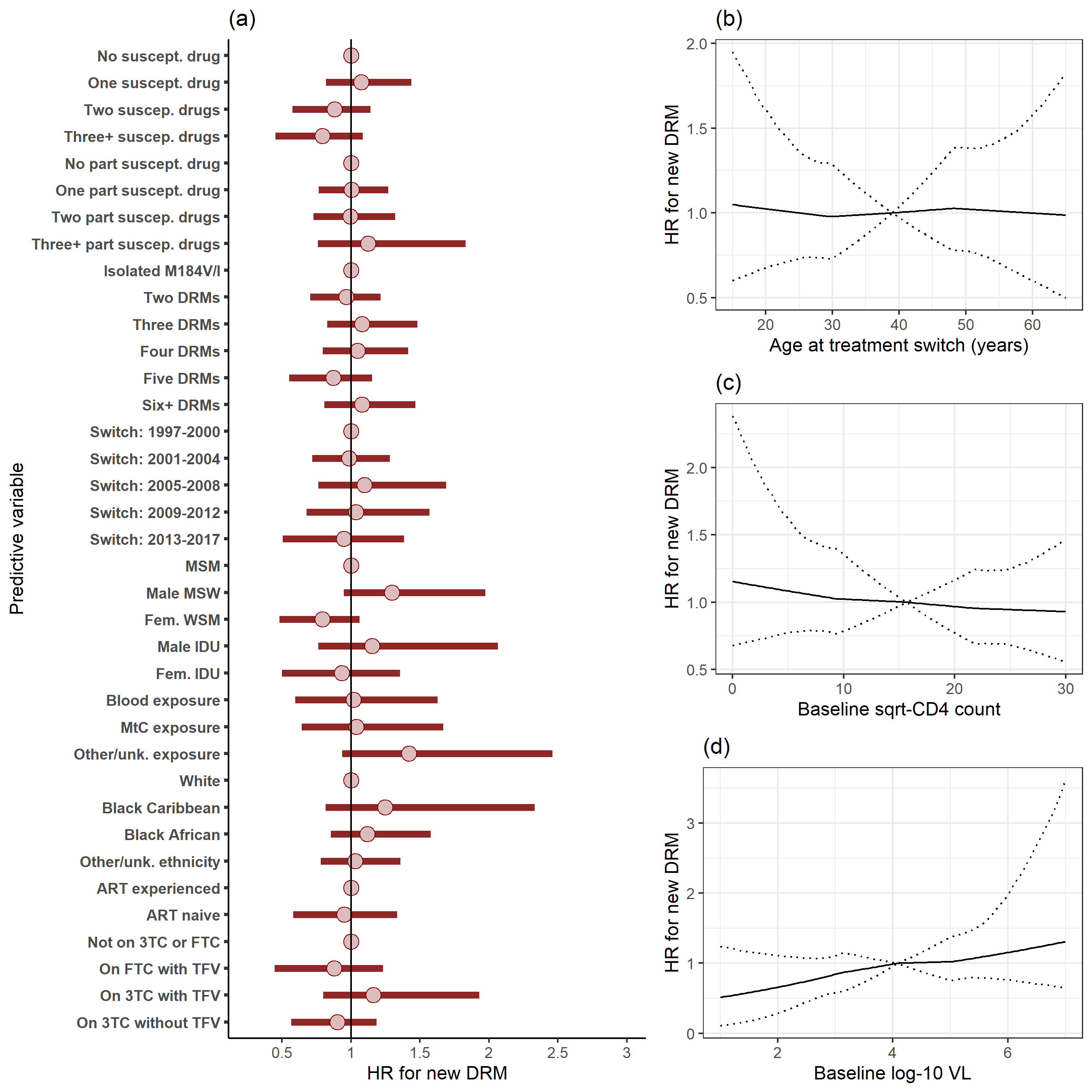


3TC, lamivudine; ART, antiretroviral therapy; DRM, major drug resistance mutation; FTC, emtricitabine; IDU, injecting drug user; MSM, men who have sex with men; MSW, men who have sex with women; MtC, mother-to child; TFV, tenofovir; unk., unknown; VL, viral load; WSM, women who have sex with men.

**Figure S8**  Associations between individual, viral and ART characteristics and incidence rate of new viral DRM following ART switch subsequent to detection of the M184V/I mutation. Incidence rate ratios (IRR) estimated through a Bayesian implementation of a Poisson model conditional on ART combination (±3TC/FTC) and with person-specific frailty term and random effects for clinical centre. Categorical variables are shown in (a), with reference groups displayed as a fixed value of ‘1’. Associations between continuous variables of baseline (b) age, (c) CD4 count and (d) VL and incidence of new DRM are shown separately. Estimates are shown as posterior mean and 95% credibility interval.


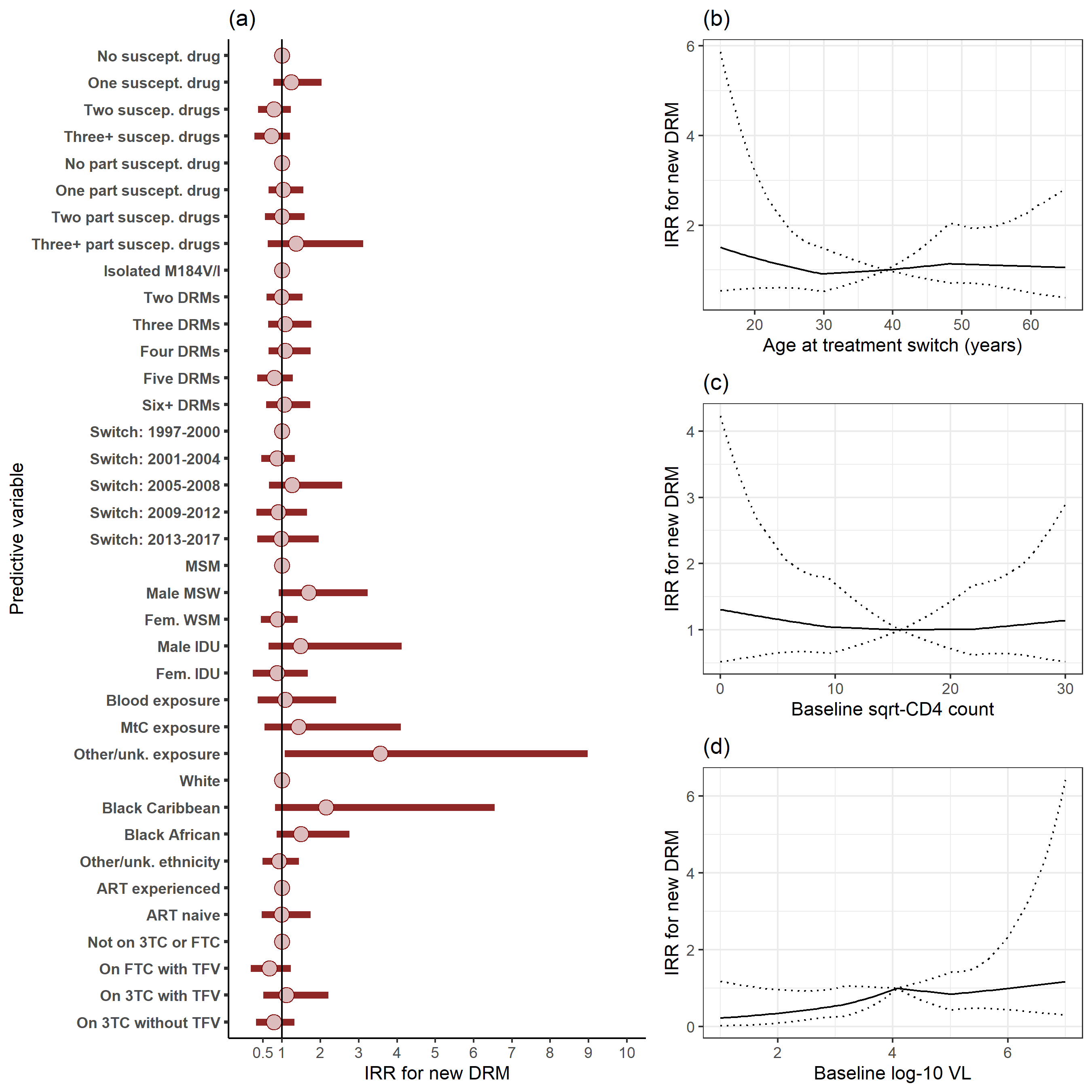


3TC, lamivudine; ART, antiretroviral therapy; DRM, major drug resistance mutation; FTC, emtricitabine; IDU, injecting drug user; MSM, men who have sex with men; MSW, men who have sex with women; MtC, mother-to child; TFV, tenofovir; unk., unknown; VL, viral load; WSM, women who have sex with men.

**Figure S9** Associations between individual, viral and ART characteristics and incidence rate of new viral DRMs following ART switch subsequent to detection of the M184V/I mutation, with separate effect estimates for 3TC or FTC use before or after 2007. Incidence rate ratios (IRR) estimated through a Bayesian implementation of a Poisson model conditional on ART combination (±3TC/FTC) and with person-specific frailty term and random effects for clinical centre. Categorical variables are shown in (a), with reference groups displayed as a fixed value of ‘1’. Associations between continuous variables of baseline (b) age, (c) CD4 count and (d) VL and incidence of new DRM are shown separately. Estimates are shown as posterior mean and 95% credibility interval.


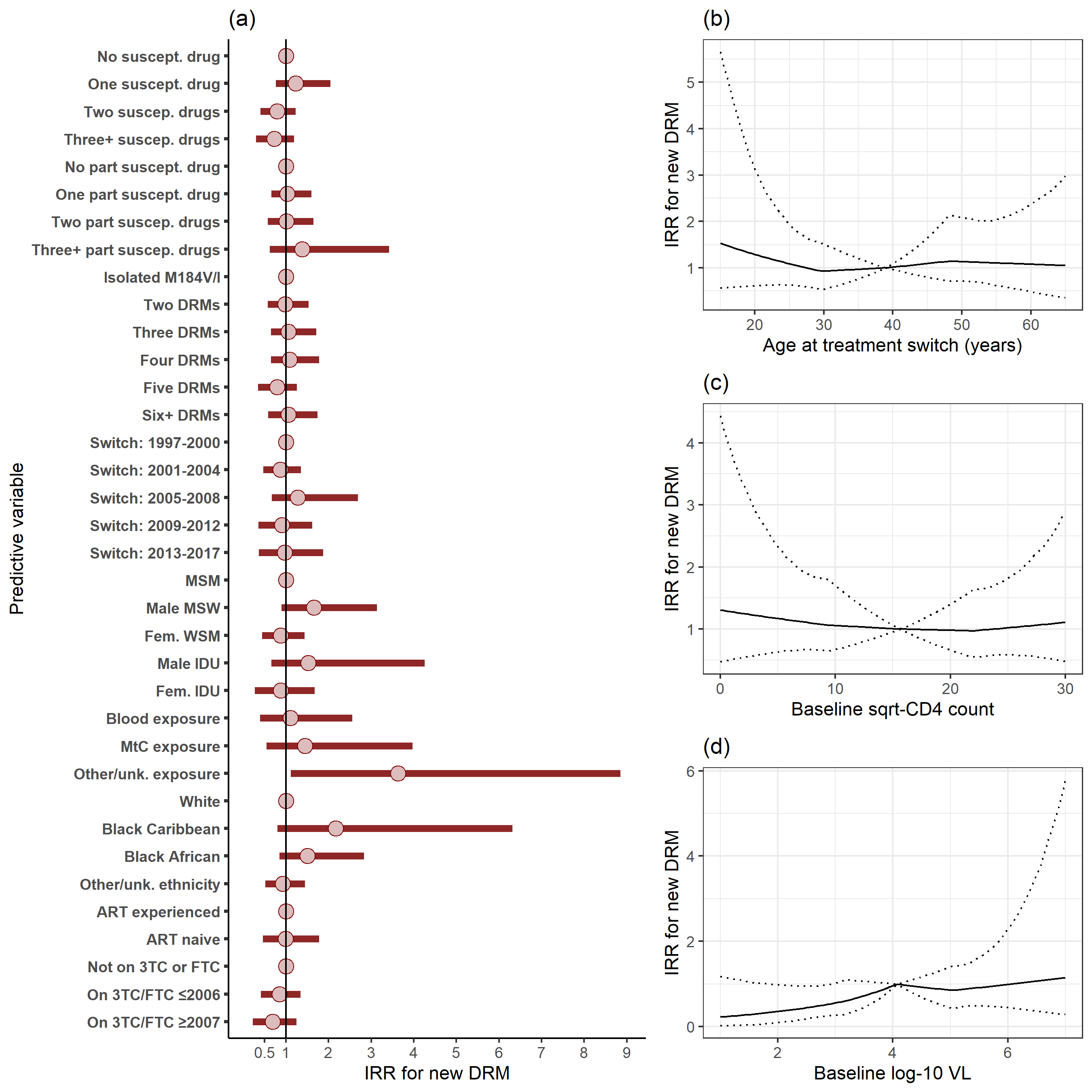


3TC, lamivudine; ART, antiretroviral therapy; DRM, major drug resistance mutation; FTC, emtricitabine; IDU, injecting drug user; MSM, men who have sex with men; MSW, men who have sex with women; MtC, mother-to child; unk., unknown; VL, viral load; WSM, women who have sex with men.
